# Transcriptome Analyses Indicate Significant Association of Increased Non-Additive and Allele-Specific Gene Expression with Hybrid Weakness in Rice (*Oryza sativa* L.)

**DOI:** 10.3390/life12081278

**Published:** 2022-08-21

**Authors:** Yingheng Wang, Jing Xia, Likun Huang, Qiang Lin, Qiuhua Cai, Hongguang Xie, Wei He, Yidong Wei, Huaan Xie, Weiqi Tang, Weiren Wu, Jianfu Zhang

**Affiliations:** 1 Rice Research Institute, Fujian Academy of Agricultural Sciences, Fuzhou 350019, China; 2 National Rice Engineering Research Center of China, Fuzhou 350003, China; 3 State Key Laboratory of Ecological Pest Control for Fujian and Taiwan Crops, Fuzhou 350003, China; 4 Incubator of National Key Laboratory of Fujian Germplasm Innovation and Molecular Breeding between Fujian and Ministry of Science and Technology of China, Fuzhou 350003, China; 5 Base of South China, State Key Laboratory of Hybrid Rice, Fuzhou 350003, China; 6 Key Laboratory of Germplasm Innovation and Molecular Breeding of Hybrid Rice for South China, Ministry of Agriculture and Rural Affairs of China, Fuzhou 350003, China; 7 Fuzhou Branch, National Rice Improvement Center of China, Fuzhou 350003, China; 8 Fujian Engineering Laboratory of Crop Molecular Breeding, Fuzhou 350003, China; 9 Fujian Key Laboratory of Rice Molecular Breeding, Fujian Academy of Agricultural Sciences, Fuzhou 350003, China; 10 College of Agriculture, Fujian Agriculture and Forestry University, Fuzhou 350002, China; 11 Marine and Agricultural Biotechnology Laboratory, Fuzhou Institute of Oceanography, Minjiang University, Fuzhou 350108, China

**Keywords:** mid-parent hybrid weakness (MPHW), non-additive gene expression (NAE), allele-specific gene expression (ASE), transcriptome

## Abstract

The heterosis in hybrid rice is highly affected by the environment and hybrid weakness occurs frequently depending on the genotypes of the hybrid and its parents. Hybrid weakness was also observed in our field experiments on nine rice hybrids produced by 3 × 3 incomplete diallel crosses. Among the nine hybrids, five displayed mid-parent heterosis (MPH) for grain yield per plant, while four showed mid-parent hybrid weakness (MPHW). A sequencing analysis of transcriptomes in panicles at the seed-filling stage revealed a significant association between enhanced non-additive gene expression (NAE) and allele-specific gene expression (ASE) with hybrid weakness. High proportions of ASE genes, with most being of mono-allele expression, were detected in the four MPHW hybrids, ranging from 22.65% to 45.97%; whereas only 4.80% to 5.69% of ASE genes were found in the five MPH hybrids. Moreover, an independence test indicated that the enhancements of NAE and ASE in the MPHW hybrids were significantly correlated. Based on the results of our study, we speculated that an unfavorable environment might cause hybrid weakness by enhancing ASE and NAE at the transcriptome level.

## 1. Introduction

Rice (*Oryza sativa*) is an important cereal food worldwide. The success and application of three-line hybrid rice in the 1970s contributed to the food security of China [1] and the world. Elite hybrid rice varieties, such as Shanyou 63, were widely planted in Southeast Asia and in other regions. Rice is also a model plant for crop research. Rice growth and development involves complex and delicate regulatory systems. To further understand the mechanisms of heterosis in rice, it is necessary to dissect the differences in gene expression and regulation among parents and hybrids at the genome-wide level. Allelic variations between rice parental lines and the modified global gene expression levels in highly heterozygous hybrids may be responsible for hybrids’ vigor [2].

**Figure 1:**
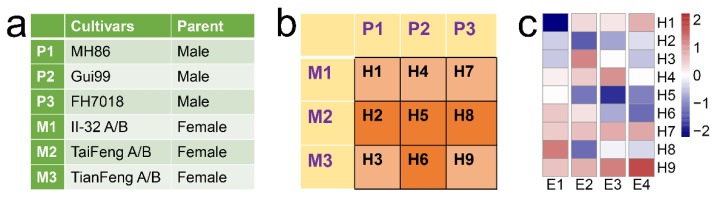
Results of field experiments. (**a**) Parents of three-line hybrid rice used for crosses. The female parents are cytoplasmic male sterile lines (A) and corresponding maintainer lines (B); the male parents are restorer lines. (**b**) Experimental design of 3 × 3 incomplete diallel cross. (**c**) Mid-parent heterosis (MPH) values of nine hybrids on grain yield per plant (GYPP) under four environments (E1–E4). The color (red to blue) of the heat map represents the column-scaled MPH.

Heterosis is very useful for agricultural production. Hybrid rice has been making great contributions to the increase of rice yield in China and other countries. As a complex trait, however, heterosis is also influenced by the environment. A hybrid may exhibit heterosis in some environments, but show hybrid weakness in others, which is an opposite phenomenon of heterosis. For example, the phenomenon of temperature-dependent hybrid weakness has been observed in some species such as rice [3,4] and *capsicum* [5]. Hybrid weakness was also observed by our field experiment with nine rice hybrids (H1–H9) from 3 × 3 incomplete diallel crosses (Figure 1a,b) conducted in four different environments across two locations and three years, including Fujian 2016 (E1), Hainan 2016 (E2), Hainan 2017 (E3), and Hainan 2019 (E4). The results showed that only two hybrids (H7 and H9) exhibited mid-parent heterosis (MPH) on grain yield per plant (GYPP) in all four environments (Table S1), while the other hybrids displayed either little MPH or even mid-parent hybrid weakness (MPHW) in one or more environments (Figure 1c), suggesting that the heterosis in hybrid rice is highly affected by environment and hybrid weakness occurs frequently, depending on the genotypes of the hybrid and its parents.

The genetic mechanisms underlying heterosis have been studied for decades [2,6,7,8,9,10,11,12], but there are few studies on the molecular mechanism of hybrid weakness. The identification of non-additive expression (NAE) and allele-specific expression (ASE) patterns at the gene-level is usually used to reveal the molecular mechanism of heterosis [9,10,11]. Some hybrid-weakness-associated genes have been mapped in rice [13,14,15,16,17], but there are still no reports on the study of molecular mechanisms of hybrid weakness from the perspectives of NAE and ASE. To study the molecular mechanisms underlying hybrid weakness, we performed RNA-seq with three biological replicates of the panicles at the grain-filling stage of all the hybrid and parental lines collected in E3, where four hybrids (H2, H5, H6, and H8) displayed MPHW in GYPP (Figure 1c). Based on the RNA-seq data, we found that NAE and ASE of genes were significantly associated with MPHW in rice.

## 2. Materials and Methods

### 2.1. Plant Materials and Growth Conditions

In total, nine rice hybrids were developed from all possible crosses between three restorer lines (MH86, Gui99, and FH7018; as male parents) and three CMS lines (II32, TaifengA, and TianfengA; as female parents; Figure 1a,b) at Sanya (18°40′ N, 109°71′ E), Hainan, China in 2015. The 15 lines (including parents and hybrids) were planted with 3 replicates at Sanming (26°57′ N, 117°66′ E), Fujian, China during the summer season in 2016 (E1) and at Sanya (18°40′ N, 109°71′ E), Hainan, China during the winter season in 2016 (E2), 2017 (E3), and 2019 (E4), respectively. For each hybrid, the ‘family-trio’ experimental design was adopted. Planting and field management were performed as in a previous experiment [13]. The trait GYPP was investigated in the four environments (Table S1), with five central plants measured in each plot [18].

### 2.2. Calculation of Mid-Parent Heterosis (MPH) Index

The formula used for calculating MPH index was: MPH index=(H−MP)/MP, in which H is the hybrid value and MP is the mid-parent value [19].

### 2.3. RNA Isolation, Library Construction, and Sequencing

In each line, panicles at the grain-filling stage were collected from five plants grown in E3 and mixed for RNA extractions. Three biological replicates were set. Fresh panicles were frozen in liquid nitrogen immediately after harvest, transported by dry ice, and stored at −80 ℃. RNA was isolated from approximately 10 g of frozen panicles using TRIzol Reagent (Invitrogen) and treated with RNase-free DNase Ⅰ (NEB, Hitchin, Hertfordshire, UK. to remove genomic DNA. Qualified RNA was used for library construction and sequencing. Library construction was completed using an mRNA-Seq Sample Prep Kit (Illumina, San Diego, CA, USA) in accordance with the manufacturer’s instructions. Finally, 45 RNA libraries were used for sequencing on the Illumina HiSeq X Ten platform and paired-end reads (PE150) were generated (Table S2). 

### 2.4. Analysis of RNA-Seq Data

Raw reads were first processed using the next-generation sequencing quality control software packages, FastQC [20] and MultiQC [21], and then were cleaned using BBTools [22]. After removing reads containing adapters and low-quality reads, an average of ~7.68 Gb clean reads were obtained per sample (Table S2). The Rice Nipponbare IRGSP-1.0 assembly [23] was used as the reference genome sequence, and the MSU 7.0 annotation of rice gene models were used in this study. The clean reads were aligned against the rice reference genome using HISAT2 [24]. The mapping rates of all the samples were mostly greater than 98% (Table S3). FeatureCounts [25] was used to calculate the read counts mapped to each gene model and then the expression level (FPKM value) of each gene was calculated. Genes with high confidence (HC) were filtered using the following criterion [26]: there were at least two samples with a read count per million (CPM) value > 1. The CPM values were calculated using the R package edgeR [27]. 

### 2.5. Identification of NAE Genes 

In each cross combination, a NAE gene was identified based on the following two criteria: (1) the *p*-value obtained by *t*-test < 0.05; and (2) the log_2_(fold change of H/MP) > 1. The *t*-test aimed to check the gene expression level differences between the hybrid (denoted by H) and the mid-parent, MP = (P + M)/2, where P and M are the expression levels of the male and the female parents, respectively. The *t* statistic was calculated by the following formula:

$$t=\frac{H-(P+M)/2}{\sqrt{\frac{\sigma_{H}^{2}}{n_{H}}+\frac{\sigma_{P}^{2}}{4n_{P}}+\frac{\sigma_{M}^{2}}{4n_{M}}}}$$

where $\sigma_{P}^{2}$, $\sigma_{M}^{2}$, and $\sigma_{H}^{2}$ are the variances of P, M and H, respectively; and $n_{P}$, $n_{M}$, and $n_{H}$ are the corresponding sample sizes. The degrees of freedom of the *t* statistic are: $df=\left(n_{P}-1\right)+\left(n_{M}-1\right)+\left(n_{H}-1\right)$. In this study, $n_{P}=n_{M}=n_{H}=3$ (biological replicates). Before the *t*-test, genes with low expression levels were filtered out based on the following two criteria: (1) the average expression level (FPKM value) of the gene in the 9 ($n_{P}+n_{M}+n_{H}$) samples > 1; and (2) at least in two samples the gene’s expression level ≠ 0. 

### 2.6. Identification of ASE Genes 

#### 2.6.1. Whole Genome Re-Sequencing of Parental Lines

The whole genomes of the six parental lines were re-sequenced. DNA was isolated using a plant genomic DNA extraction kit (TIANGEN) and treated with DNase-free RNaseA (NEB) to remove RNA. Qualified DNA was used for library construction. Small DNA fragment libraries (PE150, insert size of ~350 bp) were constructed and sequenced on an Illumina HiSeq X Ten platform. 

#### 2.6.2. DNA Sequencing Read Alignment and Variant Calling, Filtering, and Annotation

Raw data were first processed to eliminate adaptors and low-quality sequences using BBTools [22]. Trimmed reads (Table S4) were mapped to the reference genome of Nipponbare (IRGSP-1.0) [23] using BWA mem [28]. Samblaster [29] was used to remove PCR duplications. Alignments were sorted using Samtools [30]. Only uniquely-mapped alignments were used for later analyses (Tables S5 and S6). SNPs and InDels were called using FreeBayes [31] with the default parameters. The variant effect annotation was performed using snpEff [32]. For each cross combination, polymorphic markers with different homozygous genotypes (AA vs. aa) between two parents were identified using our custom perl scripts. The number of polymorphic SNPs varied from 663,652 to 943,338, and that of polymorphic InDels varied from 82,680 to 117,179 (Table S7).

#### 2.6.3. Detection of Exonic SNP and InDel Markers

Firstly, variants located in TE-related genes were excluded according to the MSU 7.0 genome annotation. Then, based on the results of variant effect annotation, the exonic SNPs and InDels from non-TE genes were further extracted.

#### 2.6.4. Detection of Expressed SNP and InDel Markers

RNA-seq data were re-aligned to the reference genome using BWA mem with the default parameters [28], and variant calling and joint genotyping was performed using FreeBayes [31]. The results were used to detect expressed SNPs and InDels by referring to the exonic variant set. The expressed genes with high enough sequencing depth at variant sites were saved according to the following two criteria: (1) the total depth of the three RNA-seq replicates of F_1_ > 20×; and (2) the depth in any replicate > 5×.

#### 2.6.5. Analysis of Allelic Imbalance and Detection of ASE Genes 

For each cross-combination, the parental phasing of allelic counts in F_1_ was performed at first. Then, the allelic counts of three RNA-seq replicates were merged, and the allele frequency (AF) of each expressed SNP or InDel site was calculated.

To check the significance of allelic imbalance of a marker, the *p*-value was calculated using a binomial distribution. Then, Bonferroni correction was used to find the *p*-value threshold at the overall significance level of 0.05. The SNPs or InDels with a *p*-value exceeding the significance threshold were taken as ASE sites. A gene with at least one ASE site was considered as a potential ASE gene. To identify ASE genes more accurately, the relative abundance of maternal allele expression (RAMAE) of each gene was estimated using the following approach: across the gene’s region, non-overlapped tiny blocks (size = 500 bp) were scanned to calculate the average AF value in each block, and then the mean of the average AF values of all the blocks was calculated as the estimate of RAMAE. An ASE gene was identified based on the following two criteria: (1) there was at least one ASE site in the gene; and (2) RAMAE value < 0.2 or > 0.8, which indicated paternal ASE (paternal allele expression > maternal allele expression) or maternal ASE (paternal allele expression < maternal allele expression), respectively. In particular, an ASE gene is regarded as mono-allele expression when its RAMAE < 0.02 or > 0.98.

## 3. Results

### 3.1. NAE Was Increased in the MPHW Hybrids

Among the nine hybrids collected in E3, four hybrids (H2, H5, H6, and H8) displayed MPHW, and the other five hybrids displayed MPH (Figure 1c). Transcriptome sequencing produced a total of 347 Gb of clean data (Table S2). The expression levels of genes were quantified by aligning clean data to the reference genome (Nipponbare IRGSP-1.0). The number of genes with high confidence in expression was similar in each cross, varying between 19,574–20,631, with an average of 20,171 (Table 1).

**Figure 2:**
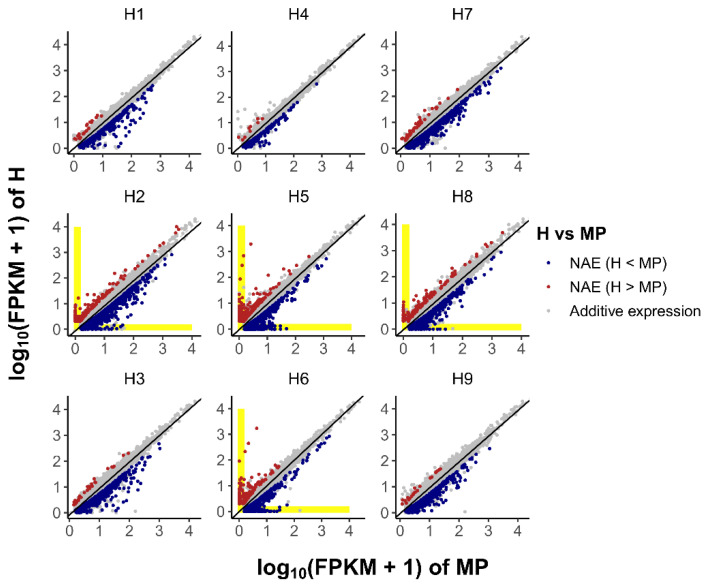
Scatter diagrams of gene expression in hybrid (H) vs. the mid-parent value (MP) showing additive expression and non-additive expression (NAE) in different crosses.

**Figure 3:**
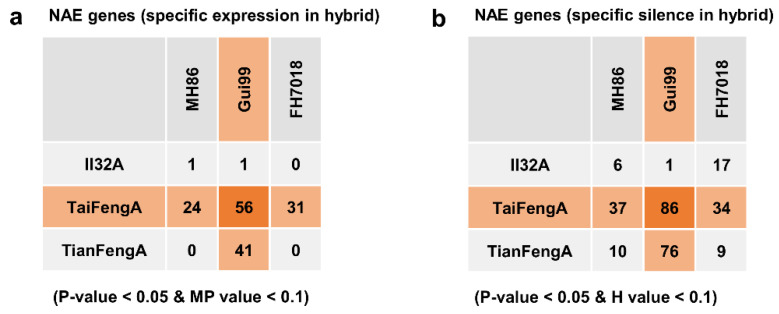
The number of NAE genes with two extreme patterns: specific expression in hybrid (**a**), and specific silence in hybrid (**b**). In parentheses at the bottom are the filter criteria.

Most genes were suggested to be additively expressed (H = MP) according to *t*-test results (Figure 2; Table 1). There were ~10% or less genes showing an NAE pattern in each hybrid, varying between 1.21–10.00%, with 1.16–9.00% downregulated (H < MP) and 0.05–1.08% upregulated (H > MP), respectively. The MPHW hybrids had a significantly higher percentage of upregulated NAE genes than the MPH hybrids, but the difference for the downregulated NAE genes between the two types of hybrids was not significant (Table 1). Nevertheless, it can be seen that there were many genes showing both an extreme upregulated NAE pattern (i.e., H > MP = 0, specifically expressed in hybrid) and extreme downregulated NAE pattern (i.e., MP > H = 0, specifically silenced in hybrid) in the MPHW hybrids, but none or very few in the MPH hybrids (Figure 2 and Figure 3). The results of the *t*-test showed that the difference between the MPHW hybrids and the MPH hybrids in the percentage of extreme NAE genes was very significant, regardless of whether it was the case of upregulated (*p*-value = 6.93 × 10^−4^) or the case of downregulated (*p*-value = 6.69 × 10^−3^). These results indicated that NAE, especially the extreme NAE, was significantly enhanced in the MPHW hybrids.

### 3.2. ASE Was Increased in the MPHW Hybrids

There were ~7000 heterozygous genes in each cross on average, varying between 5773–7972 (Table 2). These heterozygous genes were investigated for detecting ASE genes. According to the variants identified from the transcriptomic data and genomic data of the parental lines, ~33,000 SNPs and 3000 InDels located in the heterozygous genes with sufficiently-expressed allele counts were found in each hybrid on average (Table 2). In each hybrid, the RAMAE value of each variant (SNP/InDel) was calculated. 

**Figure 4:**
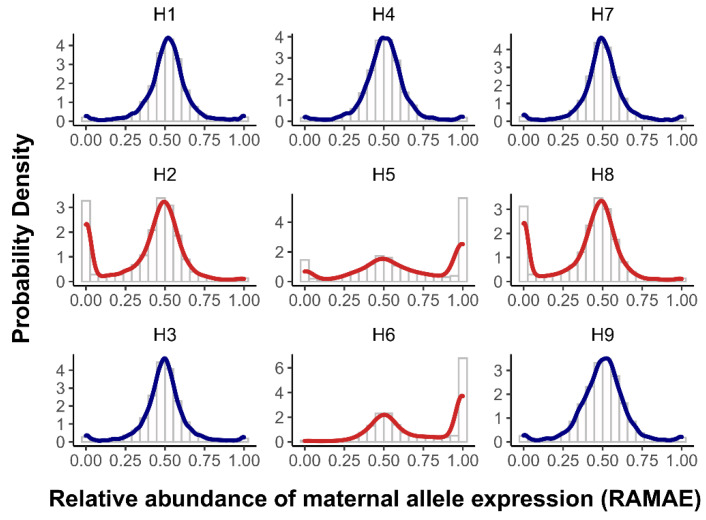
Distributions of relative abundance of maternal allele expression (RAMAE) in different hybrids.

The RAMAE approximately followed a normal distribution with the mean at the expected value of 0.5 in the MPH hybrids, except that the probability density curve slightly rose at the two ends when the RAMAE was near to 0 or 1 (Figure 4). This indicated that only a very small proportion of genes had ASE when heterosis existed. In the MPHW hybrids, the RAMAE also approximately followed a normal distribution in a wide range with the peak at 0.5, but there was an obvious extra peak at either end, indicating the existence of a large proportion of ASE genes (Figure 4). Using the above-mentioned criteria for ASE, only 351–558 ASE genes (4.80–5.69% out of total heterozygous genes, Table 3) were identified and the numbers of maternal-ASE genes and paternal-ASE genes were approximately symmetric in the MPH hybrids, while there were 1349–3095 ASE genes (22.65–45.97% out of total heterozygous genes, Table 3) found in the MPHW hybrids, with most being of mono-allele expression.

Noticeably, among the four MPHW hybrids, H2 and H8, and H5 and H6 had a similar RAMAE distribution, respectively (Figure 3). For a gene, maternal/paternal ASE means paternal/maternal allele specific silencing (ASS) and vice versa. In H2/H8, the extra peak of RAMAE distribution was at the left end, indicating that there were many genes with paternal ASE or maternal ASS. As H2 and H8 shared the same female parent, TaifengA (Figure 1a,b), the maternal ASS phenomenon appeared to be a feature of TaifengA. In contrast, the extra peak of RAMAE distribution in H5/H6 was at the right end, indicating that there were many genes with maternal ASE or paternal ASS. Likewise, H5 and H6 shared the same male parent, Gui99 (Figure 1a,b). Given this, the paternal ASS was likely to be a feature of Gui99. Interestingly, H5 shared the same female parent with H2 and H8 (Figure 1a,b), but its RAMAE distribution was similar to that of H6 rather than H2/H8. Nonetheless, different from H6, H5 had a small peak of RAMAE distribution at the left end, indicating the existence of some maternal ASS genes like that in H2/H8 (Figure 1c). Given this, it appears that the maternal (TaifengA) ASS and the paternal (Gui99) ASS occurred simultaneously in H5, but these two types of opposite events “competed” in the hybrid, with the latter being predominant. 

### 3.3. The Enhancements of NAE and ASE in the MPHW Hybrids Were Correlated

The above results indicated that both NAE and ASE were enhanced in the MPHW hybrids. Therefore, it would be interesting to see whether these two enhancements were correlated. By examining the independence between NAE and ASE with a Fisher’s exact test in the set of all detected heterozygous genes that were investigated for ASE in each cross, we found that there was a significant association (*p*-value < 0.05) between NAE and ASE in all the hybrids except H3 (*p*-value = 0.585), but the significance was much higher in the MPHW hybrids (*p*-value < 7.10 × 10^−8^) than in the MPH hybrids (*p*-value > 5.26 × 10^−4^; Table 4). This indicated that the enhancements of NAE and ASE in the MPHW hybrids were significantly correlated.

## 4. Discussion

The existence of different allelic transcripts in a hybrid increases the diversity of its transcriptome. Gene isoform expression and ASE cooperate to provide high diversity and complexity of gene regulation and expression [33]. As different alleles of the same gene may have different functions, the transcriptomic diversity (TD) in a hybrid is likely related to its adaptability to the environment, which is somewhat analogous to the genetic diversity in a species. The higher the TD is, the stronger the adaptability will be. Obviously, a gene will have the maximum diversity when its two different alleles are equally expressed, namely, the frequency of each allele in the transcriptome is 0.5. Therefore, ASE can reduce TD. This implies that a hybrid with more frequent occurrence of ASE is likely to have a lower environmental adaptability. Since ASE is significantly associated with MPHW, it is likely that hybrid weakness is a result of TD reduction. When the environment is unsuitable for the hybrid, the TD will decrease due to the increase of ASE, leading to hybrid weakness. 

In addition, for a gene with ASE, the reduced expression of an allele in the hybrid would usually be compensated by the increased expression of the other allele so that the expression level of the gene remains unchanged. However, if the compensation cannot meet the reduction, the expression level of the gene in the hybrid will deviate (more) from the mid-parent value so as to result in NAE or increase the degree of NAE. In short, ASE can cause or intensify NAE. This explains why ASE and NAE are correlated.

Taken together, we can draw a primary and rough picture for the mechanism underlying hybrid weakness at the transcriptome level in rice, that is, environment → allele-specific expression → non-additive expression → hybrid weakness. 

Among the six parental lines, MH86, Gui99, MH63, and II-32A have been widely used in China’s three-line hybrid rice breeding. However, their offspring showed different yield heterosis performance. II-32A is a superior CMS line that was used to derive the highest number of hybrids in China’s hybrid breeding history. Genes in the cross II-32A × Gui99 appear to be harmoniously expressed in the hybrid and not easily affected by the environment. In future breeding work, selecting and utilizing such materials as paternal lines will make it easy to obtain rice varieties with heterosis.

## 5. Conclusions

In conclusion, our study showed that NAE and ASE were significantly enhanced in the MPHW hybrids, and the enhancements of NAE and ASE were significantly correlated. The results suggest that NAE and ASE are associated with hybrid weakness. It is possible that an unfavorable environment causes hybrid weakness by enhancing ASE and NAE at the transcriptome level. 

## Figures and Tables

**Table 1:** Number of non-additively expressed (NAE) genes detected in each cross.

Hybrid	Type	HC Genes ^a^	NAE Genes ^b^				
			H > MP	%	H < MP	%	Total %
H1	MPH	19,962	24	0.12	433	2.17	2.29
H2	MPHW	20,257	202	1.00	1823	9.00	10.00
H3	MPH	20,109	30	0.15	726	3.61	3.76
H4	MPH	19,574	9	0.05	227	1.16	1.21
H5	MPHW	20,041	216	1.08	659	3.29	4.37
H6	MPHW	20,048	156	0.78	439	2.20	2.98
H7	MPH	20,490	64	0.31	1261	6.15	6.46
H8	MPHW	20,631	167	0.81	804	3.90	4.71
H9	MPH	20,601	25	0.12	591	2.87	2.99
Mean ± SD	MPH			0.15 ± 0.10		3.19 ± 1.88	
	MPHW			0.92 ± 0.15		4.60 ± 3.02	
*p*-value				5.77 × 10^−5^		0.428	

Note: ^a^ Genes with high confidence in expression in both parents and the hybrid. ^b^ Estimated from the *t*-test (two-tail) of the difference between MPH and MPHW, for which the percentage data were converted with the formula $\arcsin\sqrt{x}$.

**Table 2:** Number of variants (SNPs and InDels) and heterozygous genes for ASE analysis.

Crosses	SNP		InDel		Total Genes
	Variants	Genes	Variants	Genes	
H1	32,325	7122	3312	2195	7366
H2	25,535	5570	2596	1715	5773
H3	27,753	6433	2847	1875	6648
H4	34,962	7111	3479	2210	7308
H5	32,953	6472	3363	2108	6657
H6	33,052	6807	3380	2148	7015
H7	38,979	7557	3932	2474	7788
H8	32,465	6385	3200	2043	6574
H9	35,434	7233	3543	2259	7449

**Table 3:** Identification of ASE genes.

Crosses	Het. Genes	Maternal-ASE Genes		Paternal-ASE Genes		ASE Genes
		Number	Rate	Number	Rate	
H1	7366	205	2.78%	188	2.55%	393
H2	5773	116	2.01%	**1233**	**21.36%**	**1349**
H3	6648	165	2.48%	213	3.20%	378
H4	7308	178	2.44%	173	2.37%	351
H5	6657	**2367**	**35.56%**	**693**	**10.41%**	**3060**
H6	7015	**2984**	**42.54%**	111	1.58%	**3095**
H7	7788	194	2.49%	232	2.98%	426
H8	6574	141	2.14%	**1348**	**20.51%**	**1489**
H9	7449	182	2.44%	222	2.98%	404

Note: Bold fonts indicate that these results (Number and Rate) were beyond expectations.

**Table 4:** The independence test of two categorical gene sets (ASE genes and NAE genes).

Crosses	ASE	Non-Additive Expressed		*p*-Value
		TRUE	FALSE	
H1	TRUE	14	317	0.000526
	FALSE	98	6836	
H2	TRUE	140	1157	1.56 × 10^−8^
	FALSE	262	4122	
H3	TRUE	6	316	0.585
	FALSE	161	6056	
H4	TRUE	5	302	0.029
	FALSE	36	6877	
H5	TRUE	119	2814	7.10 × 10^−8^
	FALSE	66	3557	
H6	TRUE	100	2855	1.10 × 10^−10^
	FALSE	44	3897	
H7	TRUE	30	322	0.0008
	FALSE	313	6986	
H8	TRUE	86	1322	1.20 × 10^−10^
	FALSE	122	4917	
H9	TRUE	14	330	0.0023
	FALSE	112	6857	

## Data Availability

Not applicable.

## Supplementary Materials

The following supporting information can be downloaded at: https://www.mdpi.com/article/10.3390/life12081278/s1, Table S1: GYPP field performance of parental lines and hybrids; Table S2: Statistics of RNA sequencing data; Table S3: The mapping rate of RNA-seq reads (mapped by using HISAT2); Table S4: Statistics of DNA sequencing data; Table S5: The mapping rate of whole genome sequencing reads (mapped by using BWA MEM); Table S6: The coverage of whole genome sequencing reads (mapped by using BWA MEM); Table S7: Markers with different homozygous genotypes between two parents.
