# Supplementary material for: Transcriptome Analyses Indicate Significant Association of Increased Non-Additive and Allele-Specific Gene Expression with Hybrid Weakness in Rice (Oryza sativa L.)
Source: Life (Basel). 2022 Aug 21;12(8):1278. doi: 10.3390/life12081278 (PMC9410013; doi:10.3390/life12081278)
Supplement: Supplementary file 1 [file life-12-01278-s001.zip › life-1834012-supplementary.pdf]

Supplementary Tables

Table S1. GYPP field performance of parental lines and hybrids.

Table S2. Statistics of RNA sequencing data.

Table S3. The mapping rate of RNA-seq reads (mapped by using HISAT2).

Table S4. Statistics of DNA sequencing data.

Table S5. The mapping rate of whole genome sequencing reads (mapped by using BWA MEM).

Table S6. The coverage of whole genome sequencing reads (mapped by using BWA MEM).

Table S7. Markers with different homozygous genotypes between two parents.

Table S1. GYPP field performance of parental lines and hybrids.

	GYPP (2016FJ)			GYPP (2016HN)			GYPP (2017HN)			GYPP (2019HN)		
	**rep1**	**rep2**	**rep3**	**rep1**	**rep2**	**rep3**	**rep1**	**rep2**	**rep3**	**rep1**	**rep2**	**rep3**
**P1 (MH86)**	39.59	42.11	37.00	28.30	26.00	19.72	21.52	38.22	37.81	39.57	41.41	39.04
**P2 (Gui99)**	33.40	34.72	32.92	26.94	18.55	20.43	23.03	32.76	31.95	32.42	25.86	34.79
**P3 (FH7018)**	37.22	31.95	42.96	31.45	29.07	26.09	20.64	28.05	28.87	45.73	28.09	29.22
**M1 (Ⅱ-32 A/B)**	33.69	42.28	32.18	32.67	22.70	24.15	22.66	28.51	33.07	40.91	40.73	33.03
**M2 (TaiFeng A/B)**	32.25	26.67	36.09	32.19	20.86	22.40	24.21	30.53	36.63	44.90	45.50	41.76
**M3 (TianFeng A/B)**	27.39	41.32	29.95	26.05	20.22	21.14	23.77	33.94	39.84	35.41	41.54	44.30
**H1 (Ⅱ-32A/MH86)**	28.60	35.91	39.80	36.68	23.85	31.37	33.82	47.20	45.29	58.58	58.67	54.72
**H4 (Ⅱ-32A/Gui99)**	40.04	50.25	44.89	35.30	30.25	23.47	38.19	51.03	48.76	50.29	42.24	43.71
**H7 (Ⅱ-32A/FH7018)**	44.40	48.19	56.34	31.17	32.37	40.09	28.86	50.79	44.64	54.12	62.22	45.54
**H2 (TaiFengA/MH86)**	34.65	45.27	48.39	24.14	16.98	24.81	44.38	29.51	33.18	45.05	56.96	55.29
**H5 (TaiFengA/Gui99)**	39.34	38.20	47.06	19.97	21.15	24.04	32.36	26.35	20.64	41.50	39.97	40.66
**H8 (TaiFengA/FH7018)**	45.87	48.67	58.30	27.02	19.92	26.44	46.83	30.75	32.19	60.31	40.66	45.42
**H3 (TianFengA/MH86)**	40.86	42.49	47.04	27.24	35.56	32.32	46.15	44.56	39.30	45.82	49.82	50.02
**H6 (TianFengA/Gui99)**	39.70	42.68	53.14	25.01	24.45	29.16	30.58	40.14	36.07	36.86	41.07	34.52
**H9 (TianFengA/FH7018)**	40.86	47.10	56.06	39.50		25.60	50.80	52.98	40.05	56.11	63.13	68.78

Table S2. Statistics of RNA sequencing data.

Sample	Raw Base(G)	Clean Base(G)	Rate(%)	Q20(%)	Q30(%)	GC (%)
MH86-1	6.58	6.5	98.83	96.6	91.67	51.94
MH86-2	7.02	6.97	99.25	96.02	90.35	52.72
MH86-3	8.41	8.34	99.09	95.66	89.7	53.59
Gui99-1	6.52	6.47	99.21	96.03	90.53	51.7
Gui99-2	7.21	7.16	99.29	96.4	91.35	51.42
Gui99-3	7.26	7.2	99.16	96.19	90.88	51.38
FH7018-1	7.6	7.47	98.39	96.76	91.97	52.18
FH7018-2	6.74	6.63	98.27	97.5	93.56	52.1
FH7018-3	8.13	8.02	98.66	95.96	90.47	52.37
II-32B-1	7.8	7.72	99	96.02	90.56	53.11
II-32B-2	8.31	8.2	98.64	96.35	91.2	53.02
II-32B-3	8.46	8.36	98.9	94.96	88.63	52.14
TaiFenB-1	7.44	7.37	98.99	95.06	88.65	51.73
TaiFenB-2	6.93	6.87	99.07	95.16	88.84	51.2
TaiFenB-3	6.59	6.52	98.96	93.79	85.88	52.52
TianFenB-1	7.51	7.38	98.29	96.03	90.6	51.41
TianFenB-2	7.44	7.36	98.93	95.85	90.23	52.72
TianFenB-3	7.56	7.49	99.13	95.95	90.42	52.22
II-32AxMH86-1	8.15	8.09	99.26	95.84	90.2	51.89
II-32AxMH86-2	7.26	7.2	99.24	95.94	90.4	51.73
II-32AxMH86-3	6.79	6.74	99.23	95.81	90.16	52.31
II-32AxGui99-1	8.35	8.26	99.02	95.31	89.14	51.69
II-32AxGui99-2	7.34	7.26	98.94	95	88.53	52.03
II-32AxGui99-3	7.38	7.32	99.15	93.91	86.1	52.01
II-32AxFH7018-1	7.12	7.06	99.15	94.28	86.73	52
II-32AxFH7018-2	6.8	6.74	99.21	94.05	86.3	51.46
II-32AxFH7018-3	6.92	6.86	99.03	95.22	88.94	51.98
TaiFenAxMH86-1	8.21	8.11	98.77	96.11	90.77	51.55
TaiFenAxMH86-2	7.67	7.57	98.79	95.94	90.4	51.95
TaiFenAxMH86-3	7.46	7.34	98.4	94.84	88.02	52.32
TaiFenAxGui99-1	8.16	8.07	98.92	94.98	88.11	51.84
TaiFenAxGui99-2	7.5	7.43	99.07	94.87	88.07	52.39
TaiFenAxGui99-3	8.53	8.46	99.17	94.9	88.12	52.26
TaiFenAxFH7018-1	7.53	7.46	99.1	94.85	88	51.97
TaiFenAxFH7018-2	7.78	7.71	99.22	94.39	87.04	52.03
TaiFenAxFH7018-3	8	7.92	98.95	94.5	87.15	52.17
TianFenAxMH86-1	9.64	9.45	98.07	94.81	87.75	52.85
TianFenAxMH86-2	9.47	9.35	98.8	94.66	87.49	51.97
TianFenAxMH86-3	8.58	8.47	98.67	94.83	87.98	53.06
TianFenAxGui99-1	8.88	8.8	99.15	94.36	87.17	52.46
TianFenAxGui99-2	7.43	7.36	99.09	94.85	88.27	51.96
TianFenAxGui99-3	7.54	7.41	98.28	94.42	87.24	52.72

Sample	Raw Base(G)	Clean Base(G)	Rate(%)	Q20(%)	Q30(%)	GC (%)
TianFenAxFH7018-1	8.82	8.73	99.05	94.56	87.55	52.12
TianFenAxFH7018-2	9.27	9.18	99.1	93.46	85.41	52.12
TianFenAxFH7018-3	7.17	7.09	98.94	94.88	88.4	52.02

Note: Q20 and Q30, the phred value was higher than 20 and 30; Phred=-10log10(e); Rate (%), the ratio of clean base and raw base.

Table S3. The mapping rate of RNA-seq reads (mapped by using HISAT2).

Sample	Total reads	Mapped<br>reads	rate (%)
**FH7018-1**	49,836,078	49,096,023	98.52%
**FH7018-2**	44,173,486	43,485,161	98.44%
**FH7018-3**	53,453,162	52,650,246	98.50%
**Gui99-1**	43,111,776	42,516,998	98.62%
**Gui99-2**	47,717,230.00	47,018,019.00	98.54%
**Gui99-3**	47,973,438	47,074,981	98.13%
**II32AxFH7018-1**	55,096,612	54,351,201	98.65%
**II32AxFH7018-2**	48,429,876	47,723,713	98.54%
**II32AxFH7018-3**	48,814,234	48,101,296	98.54%
**II32AxGui99-1**	47,048,128	46,263,902	98.33%
**II32AxGui99-2**	44,943,738	44,200,758	98.35%
**II32AxGui99-3**	45,718,144	45,150,273	98.76%
**II32AxMH86-1**	53,922,238	53,257,241	98.77%
**II32AxMH86-2**	48,020,660	47,482,406	98.88%
**II32AxMH86-3**	44,950,238	44,392,361	98.76%
**II32B-1**	51,471,052	50,745,084	98.59%
**II32B-2**	54,650,672	53,990,607	98.79%
**II32B-3**	55,760,296	54,914,767	98.48%
**MH86-1**	43,353,354	42,480,610	97.99%
**MH86-2**	46,455,288	45,899,573	98.80%
**MH86-3**	55,593,602	54,800,349	98.57%
**TaiFenAxFH7018-1**	49,715,800	49,080,363	98.72%
**TaiFenAxFH7018-2**	51,430,950	50,401,476	98.00%
**TaiFenAxFH7018-3**	52,765,974	51,988,446	98.53%
**TaiFenAxGui99-1**	53,792,208	53,017,331	98.56%
**TaiFenAxGui99-2**	49,505,602	48,789,235	98.55%
**TaiFenAxGui99-3**	56,405,592	55,648,992	98.66%
**TaiFenAxMH86-1**	54,088,922	53,480,665	98.88%
**TaiFenAxMH86-2**	50,487,428	49,936,008	98.91%
**TaiFenAxMH86-3**	48,951,012	48,334,284	98.74%
**TaiFenB-1**	49,122,822	48,052,593	97.82%
**TaiFenB-2**	45,777,272	44,988,362	98.28%
**TaiFenB-3**	43,475,540	42,489,977	97.73%
**TianFenAxFH7018-1**	58,228,208	57,430,536	98.63%
**TianFenAxFH7018-2**	61,227,666	60,176,333	98.28%
**TianFenAxFH7018-3**	47,262,072	46,577,989	98.55%
**TianFenAxGui99-1**	58,672,368	57,706,008	98.35%
**TianFenAxGui99-2**	49,054,130	48,366,386	98.60%
**TianFenAxGui99-3**	49,377,016	48,639,125	98.51%
**TianFenAxMH86-1**	63,023,262	60,783,817	96.45%
**TianFenAxMH86-2**	62,353,396	61,541,678	98.70%

Sample	Total reads	Mapped<br>reads	rate (%)
**TianFenAxMH86-3**	56,440,648	55,701,047	98.69%
**TianFenB-1**	49,187,696	48,247,036	98.09%
**TianFenB-2**	49,084,330	48,484,168	98.78%
**TianFenB-3**	49,955,592	49,320,105	98.73%

Table S4. Statistics of DNA sequencing data.

Sample	Raw data		Trimmed data			
	Base (G)	Number	Base (G)	Rate (%)	Number	Rate (%)
FH7018	9.29	61,929,546	8.27	88.98	58,651,934	94.71
Gui99	9.69	64,602,266	8.49	87.64	60,649,882	93.88
MH86	8.86	59,083,850	7.82	88.21	55,577,580	94.07
II-32B	11.48	76,547,008	9.56	83.36	70,444,934	92.16
TaiFengB	9.66	64,415,534	8.41	87.05	60,402,274	93.77
TianFengB	10.05	67,031,984	8.82	87.72	62,933,684	93.89

Table S5. The mapping rate of whole genome sequencing reads (mapped by using BWA MEM).

Sample	Total reads	Duplicated		Mapped	
		reads	rate (%)	reads	rate (%)
**FH7018**	58,651,934	4,992,796	8.513	56,878,867	96.977
**Gui99**	60,649,882	5,710,194	9.415	58,728,356	96.832
**MH86**	55,577,580	5,252,186	9.45	53,912,459	97.004
**II32B**	70,444,934	6,985,466	9.916	66,400,012	94.258
**TaiFengB**	60,402,274	6,265,576	10.373	58,030,052	96.073
**TianFengB**	62,933,684	5,568,678	8.848	60,786,833	96.589

Table S6. The coverage of whole genome sequencing reads (mapped by using BWA MEM).

Sample	Avg. depth	1x coverage	5x coverage	7x coverage	10x coverage
FH7018	20.63	91.89%	86.73%	84.80%	81.13%
Gui99	21.64	92.34%	87.59%	85.88%	82.90%
MH86	19.92	92.97%	87.76%	85.69%	81.59%
II32B	23.58	92.68%	87.80%	86.14%	83.50%
TaiFenB	20.99	92.11%	87.12%	85.27%	81.83%
TianFenB	22.19	91.93%	86.94%	85.17%	82.18%

Table S7. Markers with different homozygous genotypes between two parents.

Cross combination	SNP number	SNP density	Ts/Tv ratio	InDel number	InDel density
**II32BxFH7018**	943,338	2.52	2.41	117,179	0.31
**II32BxGui99**	874,370	2.34	2.4	110,325	0.30
**II32BxMH86**	839,616	2.24	2.42	105,173	0.28
**TaiFengBxFH7018**	781,553	2.09	2.4	96,732	0.26
**TaiFengBxGui99**	797,956	2.13	2.4	99,207	0.27
**TaiFengBxMH86**	663,652	1.77	2.4	82,680	0.22
**TianFengBxFH7018**	895,006	2.39	2.42	109,472	0.29
**TianFengBxGui99**	821,409	2.20	2.39	103,685	0.28
**TianFengBxMH86**	756,045	2.02	2.4	95,019	0.25

Note: Density mean number of SNP or InDel markers per kb. Cross: Maternal parent x paternal parent.
